# Heart failure as a serious complication of iodinated contrast-induced hyperthyroidism: case-report

**DOI:** 10.1186/s12902-021-00870-y

**Published:** 2021-10-20

**Authors:** Sofie M. Diepenbroek, A. de Jonghe, C. van Rees, E. Seebus

**Affiliations:** 1 Department of Geriatrics, Tergooi Medical Centre, Blaricum, The Netherlands; 2 Department of Geriatrics, University Medical Centre Utrecht, Heidelberglaan 8, 3584 CS Utrecht, The Netherlands; 3 Department of Internal Medicine, Tergooi Medical Centre, Hilversum, The Netherlands

**Keywords:** Iodine-induced, ICM, Iodinated contrast media, Hyperthyroidism, Tachycardia

## Abstract

**Background:**

exposure to iodinated contrast media (ICM) can cause hyperthyroidism, due to disruption of thyroid hormone regulation. Although rare, it can have severe consequences and can lead to fatal complications. Current guidelines do not recommend standard laboratory testing of the thyroid function prior to ICM exposure. Prophylactic treatment of patients at higher risk of hyperthyroidism is not advised.

**Case presentation:**

we report the case of an 82-year-old woman who developed ICM induced hyperthyroidism after undergoing a chest computed tomography (CT). One month after ICM administration she presented with pneumonia at the emergency department. Hyperthyroidism was found with concomitant tachycardia, which was hard to control. As a result of hyperthyroidism and coincidental pneumonia the patient developed heart failure and died of myocardial ischemia.

**Conclusions:**

this case report underlines the importance of close monitoring of high-risk patients after ICM exposure. Clinicians should be aware of the risk of hyperthyroidism and potential severe complications. The use of ICM needs careful consideration, especially in the elderly who suffer more often from multinodular goitre.

## Background

There are several causes for the development of hyperthyroidism, of which the most common causes are Graves’ disease and thyroid autonomy. Exposure to iodinated contrast media (ICM) can also cause hyperthyroidism, due to disruption of thyroid hormone regulation. This is especially seen in the elderly, where multinodular goitre and accompanying thyroid autonomy are more frequently present. Although rare, ICM induced hyperthyroidism can have severe consequences [1–4]. ICM are widely used in diagnostic and therapeutic procedures such as cardiac catheterization and computed tomography (CT) [5]. Current guidelines of the Radiological Society of the Netherlands and the Netherlands Association of Internal Medicine do not recommend standard laboratory testing of the thyroid function or thyroid ultrasound investigation prior to ICM exposure [6, 7]. Prophylaxis is not recommended, although the European Society of Urogenital Radiology has concluded that it may offer some protection in high-risk patients [8]. Furthermore guidelines state that high-risk patients should be carefully monitored after administration of ICM.

Here, we aim to present a case of ICM induced hyperthyroidism that has contributed to a heart failure-related complication secondary to an ischemic event. This case study reviews the evidence that could potentially explain the physiopathology behind this case and discusses application of guidelines in daily clinical practice.

## Case presentation

**Fig. 1:**
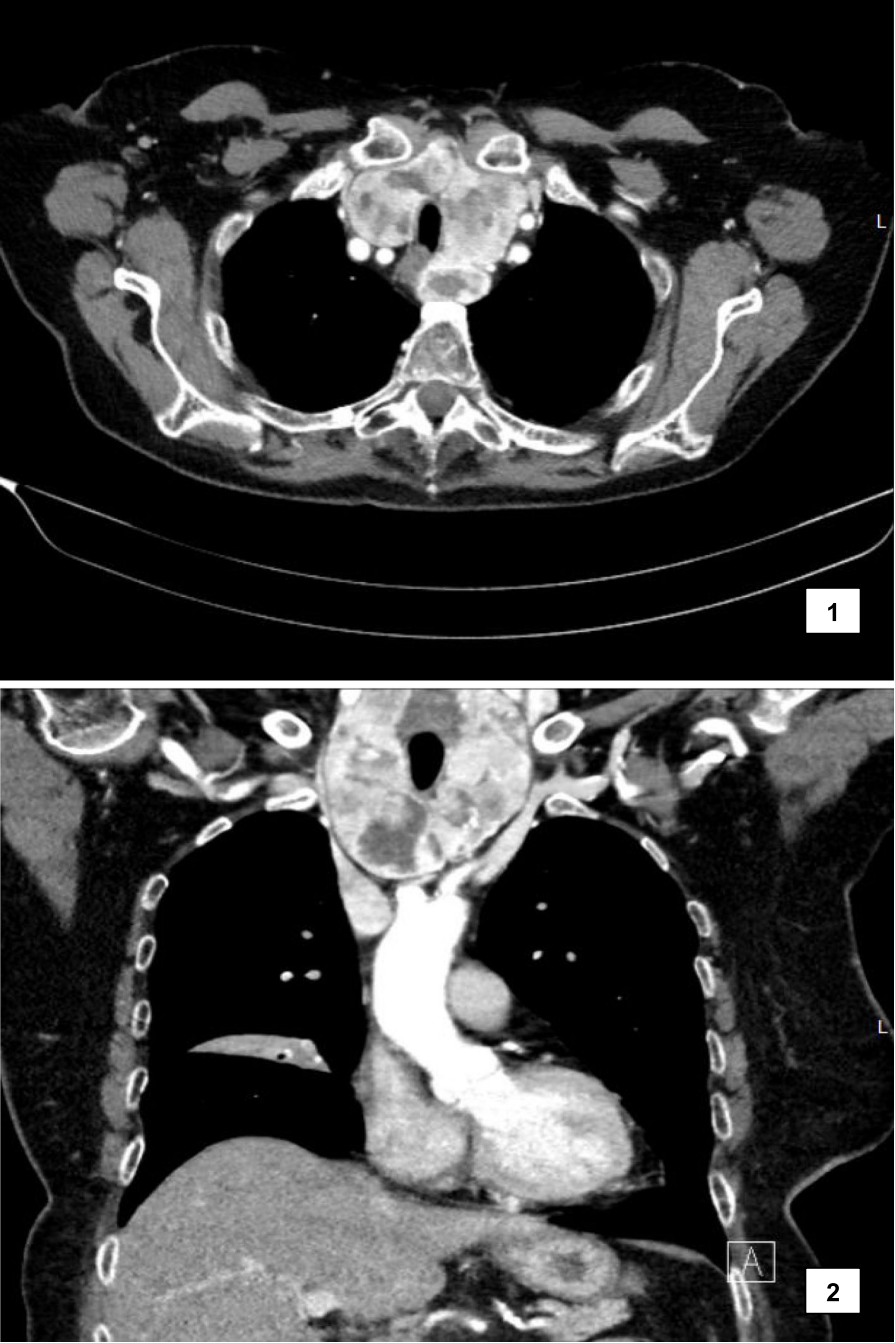
Chest CT revealing multinodular goitre; **1** transverse plane; **2** coronal plane

An 82-year-old Caucasian woman with a history of hypertension and osteoporosis was referred to a pulmonologist for outpatient diagnostic evaluation of persistent cough and signs of atelectasis on chest X-ray. A following chest CT with intravenously administered ICM (loversol 741 mg/ml) showed atelectasis and ground-glass nodules in the upper lobe of the right lung. A pulmonary function test revealed restricted lung disease, which was deemed the cause of her pulmonary complaints. Additionally, the chest CT showed a large multinodular goitre (Fig. 1). This warranted no further action at the time because the patient was asymptomatic and laboratory findings only showed subclinical hyperthyroidism.

Ten days after the chest CT, the patient was seen by her general practitioner. At that time she had some complaints of exertional dyspnoea, but was able to walk up to thirty minutes to do grocery shopping. Physical examination at that time revealed a heart rate of 84 bpm and blood pressure of 170/80 mmHg.

One month after chest CT the patient presented at the emergency department with complaints of fatigue, loss of appetite, dyspnoea and palpitations. She was not able to climb the stairs in her house and slept in a chair for two nights. Physical examination showed atrial fibrillation based tachycardia up to 160 bpm, a blood pressure of 160/70 mmHg, a saturation of 93 %, and no fever. Thyroid specific symptoms, such as myxoedema or tremor were not present. Blood tests revealed elevated inflammatory parameters, which in combination with a new consolidation in the right lung on chest X-ray pointed towards pneumonia. The blood tests also revealed a suppressed thyroid stimulating hormone (TSH) and increased T4, indicating hyperthyroidism (Table 1). The estimated glomerular filtration rate was 90 ml/min. Further patient history was negative for thyroid disease and additionally tested TSH-receptor antibody levels were below the limit of detection.

**Table 1:** Blood test results

Laboratory test	Results		
	Prior to CT	One month after CT	Reference
C-reactive protein (CRP)(mg/l)	N.D.	46	<5
leukocytes (x10^9/l)	N.D.	18.7	4-11
thyroid stimulating hormone (TSH)(mU/l)	0.22	<0.02	0.3-4.2
T4 (pmol/l)	17	38	12-22
TSH receptor antibodies (U/l)	N.D.	<0.9	<1.2

N.D.: not determined. The CRP and leucocytosis are explained by the pneumonia. Results are suggestive of an autonomously functioning multinodular goitre

**Fig. 2:**
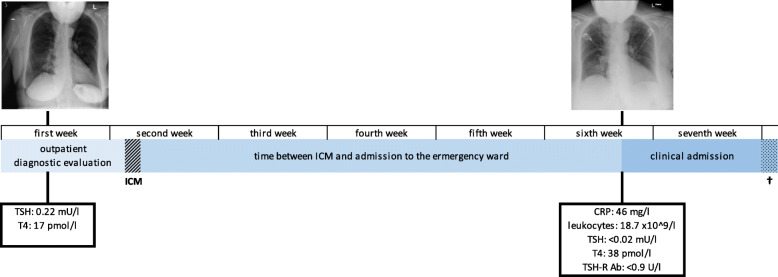
Time line. Legend: ICM = moment of chest CT with iodinated contrast media; † = time of death

The patient was admitted to the geriatric ward and antibiotic treatment for pneumonia was initiated. Prior medication (enalapril, simvastatin, budesonide/formoterol inhaler and calcium carbonate/colecalciferol) was continued with exception of alendronate which was temporarily halted. An endocrinologist was consulted and thiamazole and metoprolol were administrated to treat hyperthyroidism and tachycardia. In the following days, CRP increased from 46 mg/l on admission to 207 mg/l on day three before decreasing to 27 mg/l on day five. During admission no fever was found and blood cultures were unremarkable. A cardiologist was consulted and treatment with digoxin and apixaban was started for atrial fibrillation based tachycardia. The tachycardia appeared to be therapy resistant and the patient developed peripheral oedema. Furosemide was started, the metoprolol dose was increased and eventually verapamil was started. Also non-medication interventions were applied such as fluid restriction. Despite these interventions, little to no effect was observed. The patient persistently showed oedema, tachycardia and exertional dyspnoea. Chest X-ray was performed twice after admission and showed increasing pleural effusion with signs of cardiac decompensation. Given the limited therapeutic options, echocardiography was considered as non-contributory to improved patient outcomes by the cardiologist. The patient’s condition slowly deteriorated until eventually she was found unconscious with deep hypoxemia. Resuscitation was attempted but to no avail and the patient passed away. Autopsy revealed a right lower lobe bronchopneumonia and a hypertrophic heart with recent subendocardial ischemia in the area between the left and right coronary system. Based on the area of ischemia, hypertrophy of the left ventricle and absence of coronary artery occlusions, demand ischemia was deemed the cause of death. Most likely caused by the combination of pneumonia and persistent tachycardia Fig. 2.

## Discussion and conclusions

In retrospect, the hyperthyroidism was most likely provoked by administration of ICM during the initial chest CT and contributed to the sustained tachycardia.

In people with normal thyroids, large iodine load results in decreased synthesis of T4 and T3 due to reduced organification, halting the production of thyroid hormone. This is known as the Wolff-Chaikoff effect [9]. However, in patients with underlying thyroid disease such as multinodular goitre or Graves’ disease, this auto-regulatory process can be impaired. Although the effect of ICM on the thyroid gland is not precisely known, exposure to an excess amount of iodine can cause hypersecretion of thyroid hormones, which is known as the Jod-Basedow phenomenon [5, 10].

The prevalence of hyperthyroidism after ICM has been previously studied and the number needed to harm is as few as twenty-three [5]. Suppressed TSH levels in 6.9 % of patients four weeks after coronary angiography have been reported [11]. In patients with cardiac ischemia 7.2 % of patients have hyperthyroidism at baseline, which increases to 10 % after administration of ICM [12]. Risk factors associated with ICM induced hyperthyroidism are the presence of thyroid nodules, age over 60, male gender and a family history of thyroid disease [12]. Interestingly 4.2 % of patients still shows hyperthyroidism after three months, indicating ICM induced hyperthyroidism persists for a longer period. Although hyperthyroidism after ICM is quite common, literature regarding severity of disease and the prevalence of serious adverse events is sparse and limited to case studies [1–4]. Screening strategies and the use of prophylactic measures have been investigated. Thyroid scintigraphy with Tc-99 m has been used for risk stratification. Tc-99 m thyroid uptake has been used as a measure for the amount and function of autonomous tissue in case of TSH suppression. Patients with a Tc-99 m uptake greater than 1 % have received prophylactic treatment with perchlorate and thiamozole before undergoing coronary angiography [13]. Although effective, insight in the cost-effectiveness of such an intervention currently lacks and logistics can be challenging in emergency situations. Preventive interventions for all patients are not supported by current guidelines. Also routine TSH monitoring following ICM administration is not recommended. On the other hand, guidelines do recommend monitoring of high-risk patients after receiving ICM in order to initiate therapy if necessary. It is also stated that it is important for clinicians to mention a patient’s history of thyroid disease when ordering radiology examinations. [6–8]

In our case radiological examination with chest CT using ICM was justified, since a prior chest X-ray showed atelectasis involving the right middle lobe, which could have been the result of an obstructive lung tumor. However, the patient was at risk of developing ICM induced hyperthyroidism because of the multinodular goitre. As an elderly patient she was probably more at risk of cardiovasculair diseases as well. Atrial fibrillation is a known complication of severe hyperthyroidism, which in this case was further exacerbated by pneumonia. Excess thyroid hormones increase the amount of β-adrenergic receptors in myocardium and therefore adrenergic hyperactivity occurs, resulting in tachycardia. Additionally, free T3 stimulates the renin angiotensin aldosterone system leading to higher blood pressure and increased cardiac output [14]. The administration of a β-blocker, antithyroid drug and antibiotics in this case could not prevent cardiac decompensation and eventual demand ischemia resulting in death.

Monitoring of thyroid hormones after ICM administration should have taken place in order to treat hyperthyroidism timely, given that the patient met the definition of a high-risk patient due to multinodular goitre.

In conclusion ICM induced hyperthyroidism is a serious adverse complication that can evolve into severe heart failure, especially within the elderly with coexisting morbidities. Although the prevalence of serious adverse events is presumably limited, this case highlights the importance of monitoring high-risk patients.

## Data Availability

Not applicable.

## Abbreviations

ICM: iodinated contrast media
CT: computed tomography
TSH: thyroid stimulating hormone
